# Expression of Concern: Servant leadership and employee prosocial rule-breaking: The underlying effects of psychological safety and compassion at work

**DOI:** 10.1371/journal.pone.0329183

**Published:** 2025-07-30

**Authors:** The PLOS One Editors

The *PLOS One* Editors issue this Expression of Concern to note that concerns have been raised about the article’s compliance with the PLOS Data Availability and Authorship policies.

In addition, documentation supporting ethical approval for this study was not provided upon editorial request.
