# Supplementary material for: Servant leadership and employee prosocial rule-breaking: The underlying effects of psychological safety and compassion at work
Source: PLoS One. 2023 Apr 25;18(4):e0282832. doi: 10.1371/journal.pone.0282832 (PMC10128937; doi:10.1371/journal.pone.0282832)
Supplement: S1 File — (DOCX) [file pone.0282832.s001.docx]

Naqib Skewness and Kurtosis results

| **Descriptive Statistics** | | | | | | | | | |
| --- | --- | --- | --- | --- | --- | --- | --- | --- | --- |
|  | N | Minimum | Maximum | Mean | Std. Deviation | Skewness | | Kurtosis | |
|  | Statistic | Statistic | Statistic | Statistic | Statistic | Statistic | Std. Error | Statistic | Std. Error |
| Q1, I break org rules to do my job more efficiently | 273 | 3.00 | 5.00 | 3.6227 | .67557 | .627 | .147 | -.684 | .294 |
| Q2,I violate organizational policies to save the company time and money | 273 | 2.00 | 4.00 | 3.3626 | .48922 | .480 | .147 | -1.529 | .294 |
| Q3, I ignore organizational rules to ‘‘cut the red tape’’ and be a more effective worker | 273 | 2.00 | 3.00 | 2.8608 | .34678 | -2.096 | .147 | 2.412 | .294 |
| Q4, When organizational rules interfere with my job duties, I break those rules | 273 | 2.00 | 4.00 | 3.3333 | .47999 | .610 | .147 | -1.365 | .294 |
| Q5, I diobey company regulations that results for inefficiency of the company | 273 | 3.00 | 4.00 | 3.3993 | .49065 | .414 | .147 | -1.842 | .294 |
| Q6, I break organizational rules if my coworkers need help with their duties | 273 | 1.00 | 3.00 | 1.9780 | .75212 | .036 | .147 | -1.226 | .294 |
| Q7, When another employee needs my help, I disobey organizational policies to help him/her | 273 | 1.00 | 3.00 | 1.9158 | .67808 | .104 | .147 | -.818 | .294 |
| Q8, I assist other employees with their work by breaking organizational rules | 273 | 1.00 | 3.00 | 2.0000 | .70711 | .000 | .147 | -.989 | .294 |
| Q9, I help out other employees, even if it means disregarding organizational policies | 273 | 1.00 | 3.00 | 2.2491 | .68348 | -.362 | .147 | -.851 | .294 |
| Q10, I break rules that stand in the way of good customer service | 273 | 2.00 | 3.00 | 2.9048 | .29408 | -1.773 | .147 | 2.732 | .294 |
| Q11, I give good service to clients or customers by ignoring organizational policies that interfere with my job | 273 | 2.00 | 3.00 | 2.9158 | .27827 | -1.010 | .147 | 1.113 | .294 |
| Q12, I break organizational rules to provide better customer service | 273 | 2.00 | 3.00 | 2.9231 | .26696 | -2.193 | .147 | 2.256 | .294 |
| Q13, I bend organizational rules so that I can best assist Customers | 273 | 2.00 | 4.00 | 2.9304 | .26897 | -1.817 | .147 | 2.673 | .294 |
| Qps14, In my work unit, I can express my true feelings regarding my job | 273 | 3.00 | 5.00 | 4.2381 | .61693 | -.199 | .147 | -.570 | .294 |
| Qps15, In my work unit, I can freely express my thoughts. | 273 | 3.00 | 5.00 | 3.9744 | .43225 | -.142 | .147 | 2.402 | .294 |
| Qps16, 3In my work unit, expressing your true feelings is welcomed | 273 | 3.00 | 5.00 | 3.7143 | .46855 | -.736 | .147 | -.884 | .294 |
| Qps17, Nobody in my unit will pick on me even if I have different opinions. | 273 | 2.00 | 4.00 | 3.1612 | .44930 | .666 | .147 | .909 | .294 |
| Qps18, I’m worried that expressing true thoughts in my workplace would do harm to myself (reverse-coded). | 273 | 1.00 | 3.00 | 2.5788 | .59581 | -1.096 | .147 | .193 | .294 |
| Qsl19, My leader can tell if something work-related is going wrong. | 273 | 1.00 | 5.00 | 3.8425 | .72818 | -.266 | .147 | .226 | .294 |
| Qsl20, My leader makes my career development a priority. | 273 | 1.00 | 4.00 | 3.2564 | .47763 | .397 | .147 | .894 | .294 |
| Qsl21, I would seek help from my leader if I had a personal problem. | 273 | 1.00 | 4.00 | 2.4103 | .70724 | -.652 | .147 | -.583 | .294 |
| Qsl22, My leader emphasizes the importance of giving back to the community | 273 | 1.00 | 5.00 | 3.5165 | .60078 | -.935 | .147 | 1.799 | .294 |
| Qsl23, My leader puts my best interests ahead of his/her own. | 273 | 1.00 | 4.00 | 2.6007 | .64020 | -.594 | .147 | .148 | .294 |
| Qsl24, My leader would give me the freedom to handle diff situation the way that i feel is best | 273 | 2.00 | 5.00 | 3.1758 | .42686 | 1.256 | .147 | 1.721 | .294 |
| Qsl25, My leader would NOT compromise ethical principles in order to achieve success | 273 | 1.00 | 5.00 | 3.6300 | .54784 | -.463 | .147 | .655 | .294 |
| CAW42, I could feel compassion at work from my supervisors | 273 | 1.00 | 3.00 | 1.8315 | .54990 | -.069 | .147 | -.005 | .294 |
| CAW43, I frequently experience compassion on the job | 273 | 1.00 | 3.00 | 1.7802 | .48814 | -.457 | .147 | .008 | .294 |
| CAW44, I frequently experience compassion from my coworkers | 273 | 4.00 | 5.00 | 4.3480 | .47721 | .642 | .147 | -1.600 | .294 |
| Valid N (listwise) | 273 |  |  |  |  |  |  |  |  |

**Variable wise Skewness and Kurtosis Results**

| **Descriptive Statistics** | | | | | | | | | |
| --- | --- | --- | --- | --- | --- | --- | --- | --- | --- |
|  | N | Minimum | Maximum | Mean | Std. Deviation | Skewness | | Kurtosis | |
|  | Statistic | Statistic | Statistic | Statistic | Statistic | Statistic | Std. Error | Statistic | Std. Error |
| PSRB | 273 | 2.31 | 3.38 | 2.7997 | .24783 | .252 | .147 | -.824 | .294 |
| PS | 273 | 2.40 | 4.20 | 3.5333 | .36313 | -1.025 | .147 | .831 | .294 |
| SL | 273 | 2.29 | 3.86 | 3.2057 | .35811 | -.320 | .147 | -1.004 | .294 |
| CAW | 273 | 2.00 | 3.00 | 2.5397 | .29455 | .310 | .147 | -1.234 | .294 |
| Valid N (listwise) | 0 |  |  |  |  |  |  |  |  |

**Moderation Results SL, CW and PRSB**

Run MATRIX procedure:

***************** PROCESS Procedure for SPSS Release 2.13 ***************

Written by Andrew F. Hayes, Ph.D. www.afhayes.com

Documentation available in Hayes (2013). www.guilford.com/p/hayes3

**************************************************************************

Model = 1

Y = PSRB

X = SL

M = CW

Sample size

273

**************************************************************************

Outcome: PSRB

Model Summary

R R-sq MSE F df1 df2 p

.71 .51 .30 129.62 3.00 269.00 .00

Model

coeff se t p LLCI ULCI

constant 3.29 .04 87.63 .00 3.22 3.37

CW .63 .07 9.28 .00 .49 .76

SL .94 .10 9.00 .00 .73 1.15

int_1 .42 .19 2.17 .03 .04 .80

Interactions:

int_1 SL X CW

*************************************************************************

Conditional effect of X on Y at values of the moderator(s):

CW Effect se t p LLCI ULCI

-.58 .70 .14 4.93 .00 .42 .98

.00 .94 .10 9.00 .00 .73 1.15

.58 1.18 .16 7.24 .00 .86 1.50

Values for quantitative moderators are the mean and plus/minus one SD from mean.

Values for dichotomous moderators are the two values of the moderator.

**************************************************************************

Data for visualizing conditional effect of X on Y

Paste text below into a SPSS syntax window and execute to produce plot.

DATA LIST FREE/SL CW PSRB.

BEGIN DATA.

-.36 -.58 2.68

.00 -.58 2.93

.36 -.58 3.18

-.36 .00 2.95

.00 .00 3.29

.36 .00 3.63

-.36 .58 3.23

.00 .58 3.66

.36 .58 4.08

END DATA.

GRAPH/SCATTERPLOT=SL WITH PSRB BY CW.

******************** ANALYSIS NOTES AND WARNINGS *************************

Level of confidence for all confidence intervals in output:

95.00

NOTE: The following variables were mean centered prior to analysis:

SL CW

NOTE: All standard errors for continuous outcome models are based on the HC3 estimator

------ END MATRIX -----

**Moderation Results of SL, CW and PS**

Run MATRIX procedure:

***************** PROCESS Procedure for SPSS Release 2.13 ***************

Written by Andrew F. Hayes, Ph.D. www.afhayes.com

Documentation available in Hayes (2013). www.guilford.com/p/hayes3

**************************************************************************

Model = 1

Y = PS

X = SL

M = CW

Sample size

273

**************************************************************************

Outcome: PS

Model Summary

R R-sq MSE F df1 df2 p

.54 .29 .09 21.37 3.00 269.00 .00

Model

coeff se t p LLCI ULCI

constant 3.54 .02 186.86 .00 3.50 3.57

CW .13 .07 2.03 .04 .00 .26

SL .50 .07 7.47 .00 .37 .64

int_1 .45 .21 2.12 .03 .86 .03

Interactions:

int_1 SL X CW

*************************************************************************

Conditional effect of X on Y at values of the moderator(s):

CW Effect se t p LLCI ULCI

-.29 .64 .09 7.01 .00 .46 .81

.00 .50 .07 7.47 .00 .37 .64

.29 .37 .09 4.02 .00 .19 .55

Values for quantitative moderators are the mean and plus/minus one SD from mean.

Values for dichotomous moderators are the two values of the moderator.

**************************************************************************

Data for visualizing conditional effect of X on Y

Paste text below into a SPSS syntax window and execute to produce plot.

DATA LIST FREE/SL CW PS.

BEGIN DATA.

-.36 -.29 3.27

.00 -.29 3.50

.36 -.29 3.72

-.36 .00 3.35

.00 .00 3.54

.36 .00 3.72

-.36 .29 3.44

.00 .29 3.57

.36 .29 3.71

END DATA.

GRAPH/SCATTERPLOT=SL WITH PS BY CW.

******************** ANALYSIS NOTES AND WARNINGS *************************

Level of confidence for all confidence intervals in output:

95.00

NOTE: The following variables were mean centered prior to analysis:

SL CW

NOTE: All standard errors for continuous outcome models are based on the HC3 estimator

------ END MATRIX -----

**Mediation Results**

Run MATRIX procedure:

***************** PROCESS Procedure for SPSS Release 2.13 ***************

Written by Andrew F. Hayes, Ph.D. www.afhayes.com

Documentation available in Hayes (2013). www.guilford.com/p/hayes3

**************************************************************************

Model = 4

Y = PSRB

X = SL

M = PS

Sample size

273

**************************************************************************

Outcome: PS

Model Summary

R R-sq MSE F df1 df2 p

.51 .26 .10 95.52 1.00 271.00 .00

Model

coeff se t p LLCI ULCI

constant 1.87 .17 10.97 .00 1.54 2.21

SL .52 .05 9.77 .00 .41 .62

**************************************************************************

Outcome: PSRB

Model Summary

R R-sq MSE F df1 df2 p

.58 .34 .41 69.41 2.00 270.00 .00

Model

coeff se t p LLCI ULCI

constant -1.55 .42 -3.70 .00 -2.37 -.72

PS .62 .12 4.98 .00 .37 .86

SL .83 .13 6.64 .00 .59 1.08

************************** TOTAL EFFECT MODEL ****************************

Outcome: PSRB

Model Summary

R R-sq MSE F df1 df2 p

.53 .28 .44 104.80 1.00 271.00 .00

Model

coeff se t p LLCI ULCI

constant -.39 .36 -1.08 .28 -1.11 .33

SL 1.15 .11 10.24 .00 .93 1.38

***************** TOTAL, DIRECT, AND INDIRECT EFFECTS ********************

Total effect of X on Y

Effect SE t p LLCI ULCI

1.15 .11 10.24 .00 .93 1.38

Direct effect of X on Y

Effect SE t p LLCI ULCI

.83 .13 6.64 .00 .59 1.08

Indirect effect of X on Y

Effect Boot SE BootLLCI BootULCI

PS .32 .06 .22 .46

Partially standardized indirect effect of X on Y

Effect Boot SE BootLLCI BootULCI

PS .41 .08 .27 .58

Completely standardized indirect effect of X on Y

Effect Boot SE BootLLCI BootULCI

PS .15 .03 .10 .21

Ratio of indirect to total effect of X on Y

Effect Boot SE BootLLCI BootULCI

PS .28 .06 .17 .41

Ratio of indirect to direct effect of X on Y

Effect Boot SE BootLLCI BootULCI

PS .38 .12 .21 .70

R-squared mediation effect size (R-sq_med)

Effect Boot SE BootLLCI BootULCI

PS .17 .03 .11 .23

Preacher and Kelley (2011) Kappa-squared

Effect Boot SE BootLLCI BootULCI

PS .15 .03 .10 .20

Normal theory tests for indirect effect

Effect se Z p

.32 .07 4.42 .00

******************** ANALYSIS NOTES AND WARNINGS *************************

Number of bootstrap samples for bias corrected bootstrap confidence intervals:

1000

Level of confidence for all confidence intervals in output:

95.00

------ END MATRIX -----

**Interaction graph between SL, CW and BSRB**

**Interaction graph between SL, CW and BSRB**

**Moderated Mediation Results**

Run MATRIX procedure:

***************** PROCESS Procedure for SPSS Release 2.13 ***************

Written by Andrew F. Hayes, Ph.D. www.afhayes.com

Documentation available in Hayes (2013). www.guilford.com/p/hayes3

**************************************************************************

Model = 8

Y = PSRB

X = SL

M = PS

W = CW

Sample size

273

**************************************************************************

Outcome: PS

Model Summary

R R-sq MSE F df1 df2 p

.63 .39 .08 38.19 3.00 269.00 .00

Model

coeff se t p LLCI ULCI

constant 3.55 .02 203.12 .00 3.52 3.59

SL .44 .06 7.70 .00 .33 .56

CW .18 .03 6.15 .00 .12 .24

int_1 -.48 .09 -5.16 .00 -.66 -.29

Interactions:

int_1 SL X CW

**************************************************************************

Outcome: PSRB

Model Summary

R R-sq MSE F df1 df2 p

.73 .54 .29 115.26 4.00 268.00 .00

Model

coeff se t p LLCI ULCI

constant 1.72 .41 4.22 .00 .92 2.52

PS .44 .12 3.83 .00 .21 .67

SL .74 .12 6.02 .00 .50 .99

CW .55 .07 7.97 .00 .41 .68

int_2 .63 .19 3.34 .00 .26 1.00

Interactions:

int_2 SL X CW

******************** DIRECT AND INDIRECT EFFECTS *************************

Conditional direct effect(s) of X on Y at values of the moderator(s):

CW Effect SE t p LLCI ULCI

-.58 .38 .17 2.20 .03 .04 .72

.00 .74 .12 6.02 .00 .50 .99

.58 1.11 .16 7.13 .00 .80 1.41

Conditional indirect effect(s) of X on Y at values of the moderator(s):

Mediator

CW Effect Boot SE BootLLCI BootULCI

PS -.58 .32 .09 .17 .53

PS .00 .20 .06 .11 .33

PS .58 .07 .03 .02 .16

Values for quantitative moderators are the mean and plus/minus one SD from mean.

Values for dichotomous moderators are the two values of the moderator.

-----

Indirect effect of highest order product:

Mediator

Effect SE(Boot) BootLLCI BootULCI

PS -.21 .07 -.38 -.10

******************** INDEX OF MODERATED MEDIATION ************************

Mediator

Index SE(Boot) BootLLCI BootULCI

PS -.21 .07 -.38 -.10

******************** ANALYSIS NOTES AND WARNINGS *************************

Number of bootstrap samples for bias corrected bootstrap confidence intervals:

1000

Level of confidence for all confidence intervals in output:

95.00

NOTE: The following variables were mean centered prior to analysis:

SL CW

NOTE: All standard errors for continuous outcome models are based on the HC3 estimator

------ END MATRIX -----
