# Supplementary material for: Servant leadership and employee prosocial rule-breaking: The underlying effects of psychological safety and compassion at work
Source: PLoS One. 2023 Apr 25;18(4):e0282832. doi: 10.1371/journal.pone.0282832 (PMC10128937; doi:10.1371/journal.pone.0282832)
Supplement: Attachment — Submitted filename: Response to reviewers PONE-D-22-15473R1.docx [file pone.0282832.s002.docx]

Cover Letter of Revisions

Title: Servant Leadership and Employee Prosocial Rule-Breaking: The Underlying Effects of Psychological Safety and Compassion at Work

We are grateful to the editor for his/her interest in our work and thankful to the editor for providing their valuable comments. Comments were beneficial and constructive that had the potential of greatly increasing the contribution of this research. The paper has been revised to incorporate the editor comments. In the revised paper, the changes (additions) arising from the review are highlighted in yellow. Also, some additional changes made are incorporated to improve the paper. The following provides an explanation of the author’s response to the editor comments.

| **S.no** | **Comments** | **Responses** |
| --- | --- | --- |
| 1 | Please ensure that you refer to Table 1 in your text as, if accepted, production will need this reference to link the reader to the Table. | **R1:** Dear Sir/Madam, the reference to table 1 is cited now at line 342 in the “Confirmatory Factor Analysis” section. This change is marked yellow there, please confirm. Thanks |
| 2 | We note that your author list was updated during the revision process. Please complete our Authorship Change Form by following this link: http://journals.plos.org/plosone/s/file?id=13d0/plos-one-change-to-authorship-form.docx If you are adding or removing more than 2 authors, you can complete multiple forms and submit as many forms as needed to reflect the updates. Please return the form(s) as an attachment by emailing plosone@plos.org or by uploading it as a submission file labeled with the file type ‘Other’. Please note that if your manuscript is accepted, we will not be able to complete the publication process without the completed form. | **R2:** Dear Sir/Madam, we have previously added Prof Peng Zhongyi as a co-author because he the PhD tutor of the first and main author of the manuscript, his name is withdrawn from the manuscript now because he has not significant contribution in the manuscript due to which he himself showed willingness to withdraw his name. More, we have added two co-authors and Profs Heesup Han and Chang Tang because they both have significant contributions to the manuscript particularly during preparing the reviews on the manuscript. Moreover, we have completed the journal authorship change form which is submitted separately. Please look at the authorship change form. Thanks |
| 3 | We note your current Data Availability statement is:  - "Yes - all data are fully available without restriction" - "Data is available on request" - "Tick here if the URLs/accession numbers/DOIs will be available only after acceptance of the manuscript for publication so that we can ensure their inclusion before publication." | **R3:** Dear Sir/Madam, data is confidential and the details of confidentiality is mentioned in the “sample and procedure” section of the manuscript. But access to data can be provided only on a reasonable request by the editor from the corresponding authors. Please see at the detail answer given to the below question b. Thanks |
| PLOS journals require authors to make all data necessary to replicate their study’s findings publicly available without restriction at the time of publication. When specific legal or ethical restrictions prohibit public sharing of a data set, authors must indicate how others may obtain access to the data. For more information, please see https://journals.plos.org/plosone/s/data-availability.  Before we proceed with your manuscript, please address the following prompts: | | |
| a | If there are no restrictions, please upload the minimal anonymized data set necessary to replicate your study findings to a stable, public repository and provide us with the relevant URLs, DOIs, or accession numbers. For a list of recommended repositories, please see https://journals.plos.org/plosone/s/recommended-repositories. You also have the option of uploading the data as Supporting Information files, but we would recommend depositing data directly to a data repository if possible. | **R4:** Dear Sir/Madam, we can public the data on appropriate request by the editor, the data availability statement is added after the conclusion section at the end of the manuscript. Thanks |
| b | If there are ethical or legal restrictions on sharing a de-identified data set, please explain them in detail (e.g., data contain potentially identifying or sensitive patient information, data are owned by a third-party organization, etc.) and who has imposed them (e.g., a Research Ethics Committee or Institutional Review Board, etc.). Please also provide non-author contact information* for a data access committee, ethics committee, or other institutional body to which data requests may be sent. | **R5:** Dear Sir/Madam, during attracting respondents for participating in the surveys, we have ensured them the confidentiality or anonymity of their responses through signing the response request letters, it was necessary because we were concerned that if we did not ensure confidentiality then the respondents may report responses in a socially desirable manner about questions measuring PSRB, Psychological safety and leadership style as well, confidentiality was ensured to minimize the risk of social desirability bias, this is mentioned in detail in the “sample and procedures” section of the manuscript. Therefore if you are seeking for publically sharing the data then we must first seek respondents’ permission and ethical review board permission. We may public the data upon reasonable request from the editor. Institutional review board statement and Data availability and accessibility statements are given at the end of the manuscript before references. Thanks |

We hope that the revision is satisfactory to you. We have provided our detailed responses and specific changes to the editor Comments point-by-point in the revised manuscript.

Thank you so much.

Best regards

Naqib Ullah Khan & Co-authors
